# Direct Electron Transfer of Glucose Oxidase on Pre-Anodized Paper/Carbon Electrodes Modified through Zero-Length Cross-Linkers for Glucose Biosensors

**DOI:** 10.3390/bios13050566

**Published:** 2023-05-22

**Authors:** Gilberto Henao-Pabon, Ning Gao, K. Sudhakara Prasad, XiuJun Li

**Affiliations:** 1 Biomedical Engineering, University of Texas at El Paso, 500 W University Ave, El Paso, TX 79968, USA; 2 Independent Researcher, 206 Via Morella, Encinitas, CA 92024, USA; 3 Department of Chemistry & Biochemistry, University of Texas at El Paso, 500 W University Ave, El Paso, TX 79968, USA; 4 Yenepoya Research Centre, Yenepoya University, Mangalore 575018, Karnataka, India; 5 Forensic Science & Environmental Science and Engineering, 500 W University Ave, El Paso, TX 79968, USA

**Keywords:** microfluidic paper-based analytical device, direct electron transfer, glucose biosensors, electrochemical detection, glucose oxidase immobilization

## Abstract

A disposable paper-based glucose biosensor with direct electron transfer (DET) of glucose oxidase (GOX) was developed through simple covalent immobilization of GOX on a carbon electrode surface using zero-length cross-linkers. This glucose biosensor exhibited a high electron transfer rate (ks, 3.363 s^−1^) as well as good affinity (km, 0.03 mM) for GOX while keeping innate enzymatic activities. Furthermore, the DET-based glucose detection was accomplished by employing both square wave voltammetry and chronoamperometric techniques, and it achieved a glucose detection range from 5.4 mg/dL to 900 mg/dL, which is wider than most commercially available glucometers. This low-cost DET glucose biosensor showed remarkable selectivity, and the use of the negative operating potential avoided interference from other common electroactive compounds. It has great potential to monitor different stages of diabetes from hypoglycemic to hyperglycemic states, especially for self-monitoring of blood glucose.

## 1. Introduction

Diabetes is generally defined as a casual plasma glucose concentration that is higher than 200 mg/dL, and the incidence and prevalence of diabetes have been increasing worldwide [1]. In 2015, 9.4% of the total population of the USA, including adults and children, had diabetes (30.3 million people) [2,3]. Of those, 7.2% were diagnosed with diabetes (23.1 million people), and the rest were estimated to be undiagnosed (2.2% of the US population or 7.2 million people) [3]. As of 2015 in the US, about 5% of people diagnosed with diabetes are type 1 diabetics, and the rest are type 2 diabetics [4,5,6]. Other than diabetics, 33.9% or 84.1 million people are classified as pre-diabetes, when their biomarker glycosylated hemoglobin (HgbA_1c_) is >5.5% but <6.4% [3,7]. Pre-diabetes is defined as a condition in which individuals have fasting plasma glucose (FPG) from 100 mg/dL to 125 mg/dL (5.6–6.9 mmol/L) or HbA_1c_ from 5.7% to 6.5% (39–47 mmol/mol), or impaired glucose tolerance with plasma glucose levels measured by an oral glucose tolerance test (OGTT) that are higher than normal but not high enough to be classified as diabetes [5,8,9]. Moreover, gestational diabetes causes pregnant women to exhibit high blood glucose levels and affects 3–10% of pregnancies [9,10]. The guidelines of the American Diabetes Association (ADA) and the International Diabetes Federation (IDF) recommend that self-monitoring of blood glucose (SMBG) protocols be individualized to meet the specific needs of each patient (T1D and T2D patients) and be performed frequently during pregnancy in diabetic patients [2,11,12]. Hence, the rapid determination of glucose levels in body fluids at the point of care is a key parameter for the diagnosis and management of diabetes [9,13]. Specifically, when a patient’s glucose level is higher than 600 mg/DL, it will cause diabetic hyperosmolar syndrome, which is a life-threatening condition, and the patient needs to be quickly diagnosed and hospitalized right away.

GOX-based glucose biosensors have attracted much attention due to their simplicity in fabrication, reagent-less nature, portability, and low operational costs. However, the main limitation is that the commercially available GOX, GDH (glucose dehydrogenase), FAD (flavin-adenine-dinucleotide), and GDH (glucose dehydrogenase)-PQQ (pyrroloquinoline quinone)-based glucometers only cover the range from 20 to 600 mg/dL and cannot address all diabetes scenarios. Only two of them meet the ISO (International Organization for Standardization) 2013 criteria for the international standards for blood glucose monitoring systems [14,15]. In general, such bottlenecks arise in part due to limitations in direct electron transfer (DET) and the inaccessibility of active FAD sites (the active redox center of GOX) because FAD/FADH_2_ is deeply embedded within a protein shell at approximately a 13 Ǻ depth, which limits the electron transfer rate between the active site of GOX and the electron surface [16,17,18]. Previously, attempts at increasing the DET from GOX using functionalized carbon nanotube composites with graphene were reported. However, due to structural deformability and non-ohmic contacts, this approach suffered from low electrical conductivity or a low upper detection limit [19,20,21,22,23].

Herein, we report a new simple method for covalent immobilization of GOX on a pre-anodized paper-carbon electrode (PA-PPE) with zero-length cross-linkers for DET of glucose oxidase without utilizing any mediators and demonstrate its application in glucose biosensors. By utilizing EDC (1-ethyl-3-3-dimethylaminopropyl-carbodiimide hydrochloride) and NHS chemistry (1-Hydroxy-2,5-pyrrolidinedione or N-hydroxysuccinimide) [24,25], GOX was covalently linked to PA-PPE. This improved electrode based on the covalent binding of glucose oxidase and its cofactor FAD to a carbon electrode using short-distance co-linkers significantly improved the electron transfer rate (ks, 3.363 s^−1^) and enabled the rapid quantitation of glucose concentrations from 5.4 mg/dL to 900 mg/dL. This allowed the new DET glucose biosensor to cover the range of hypoglycemic and hyperglycemic states while maintaining the low cost and portability required for a point-of-care testing device.

## 2. Experimental Procedure

### 2.1. Materials and Reagents

Glucose oxidase from Aspergillus Niger (EC 1.1.3.4 CAS 9001-37-0), Glucose (D-(+)-glucose (>99.5%, CAS 50-99-7)), 1-ethyl-3(3-dimethylaminopropyl) carbodiimide hydrochloride (EDC) (CAS 25952-53-8), N-hydroxysulfosuccinimide (Sulfo-NHS) (CAS 106627-54-7), Ru(NH_3_) Cl_2_ (hexamine-ruthenium III chloride) (98%, CAS 14282-91-8), phosphate-buffered saline (PBS) 10X concentrate (CAS 7558-79-4) with a pH of 7.2–7.6 at 25 °C, ascorbic acid (AA) (99%, CAS 50-81-7), salicylic acid (SA) powder (CAS 69-72-7), and lactic acid (LA) (CAS 50-21-5) were all purchased from Sigma Aldrich, St. Louis, MO, USA. 

A highly concentrated graphene oxide (HC-GO) composition (≥79% carbon and ≤20% oxygen with sheet resistance 50 Ω/sq. at 25 mm thickness) was purchased from Graphene Laboratories (Calverton, NY, USA). Ag/AgCl ink (surface resistivity < 75 mΩ/square/mil and viscosity 5570–14,600 CPS) and Carbon ink 79% C-220 carbon resistive ink were obtained from Conductive Compounds. Potassium ferricyanide [K_3_Fe(CN)_6_] (CAS 13746-66-2) was purchased from Fisher Scientific. SU-8 negative photoresist formulation 50 was purchased from Micro-Chem (Newton, MA, USA). A pH 7.4 phosphate buffer solution (PBS) was used in all the studies. Deionized water (>18 MΩcm) was obtained from a Millipore purification system (Millipore, Burlington, MA, USA).

### 2.2. Electrochemical Measurements

Voltammetry measurements, cyclic voltammetry (CV), and square wave voltammetry (SWV) were carried out with a CHI 730E (CHI, Austin, TX, USA) electrochemical workstation at room temperature (25 °C). Three working electrodes, paper-carbon electrodes (PPE), graphene oxide-PPE (GO-PPE), pre-anodized-PPE (PA-PPE), and modified PA-PPE-GOX-glucose electrodes were studied with cyclic voltammetry at different scan rates ranging from 30 mV/s to 500 mV/s. 

The PA-PPE-GOX biosensor was studied in different electrochemical systems, such as 5 mM potassium ferricyanide, 5 mM hexamine ruthenium III chloride, and 0.01 M PBS. The electrochemical oxidation of the biosensor was evaluated to demonstrate the presence of immobilized GOX and, therefore, the analysis of glucose.

### 2.3. Fabrication and Modification of the Paper-Based Carbon Electrode for GOX Immobilization

**Figure 1:**
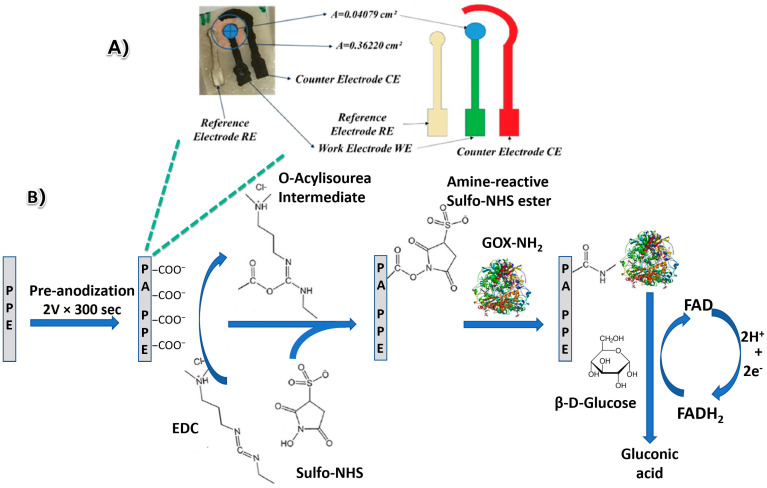
Photograph and schematic (**A**) of the paper-based glucose biosensor via direct electron transfer. The circuitry consisted of a WE, CE, and RE. (**B**) Schematic of the electrode modification process for GOX immobilization on paper-based carbon electrodes (PA-PPE-GOX) for the direct electron transfer glucose biosensor.

As shown in Figure 1A, the PPE was fabricated by pattering electrode designs onto a low-tack paper, which was subsequently pasted onto a piece of SU8-treated chromatography paper [26,27,28,29,30]. Afterwards, a carbon-based working electrode (WE area, 0.03141 cm^2^), a counter electrode (CE), and a silver pseudo reference electrode (RE) were stencil printed with conductive carbon and Ag/AgCl ink.

Figure 1B depicts the process of GOX immobilization on the electrode surface for DET glucose biosensors. The PPE electrode was prepared and subjected to pre-anodization in pH 7.4 PBS by applying a potential at 2.0 V (vs Ag/AgCl) over the electrode for 300 s. With pre-anodization, the ratio between O_1s_ and C_1s_ (O_1s_/C_1s_ ratio) changed and created more carbonyl-group functionalities [31,32]. The pre-anodization not only creates more edge plane sites, making the modified carbon electrode more electroactive but also provides low susceptibility to electrode fouling [23,33,34]. Next, 5 mL of an EDC/NHS (0.35 M/0.1 M) solution was dropped over the WE at the PA-PPE and was incubated for 30 min at room temperature. Afterwards, the device was washed with 0.01 M PBS to remove the excess. Then, GOX was coupled with the WE through EDC and NHS cross-linkers. The electrodes (PA-PPE-GOX) were then dried at room temperature for 2 h and subsequently rinsed with 0.01 M PBS to remove the unbound GOX. The prepared electrodes (PA-PPE-GOX) and modified electrodes with GO (PA-PPE-GO-GOX) were further used for the voltammetry measurements and evaluated for the electrochemical oxidation of glucose through direct electron transfer without utilizing any mediators.

### 2.4. Characterization of the Modified Electrodes

The Fourier transform infrared (FT-IR) spectra of different electrodes were recorded to verify the electrode modification using a Spectrum 100 FT-IR Spectrometer (Perkin Elmer, Waltham, MA, USA). The optical module contains a Class II/2 Helium-Neon (HeNe) laser for continuous radiation at a wavelength of 633 nm.

X-ray photoelectron spectroscopy (XPS) is a powerful technique that provides useful information on the molecular compositions and chemical bonding of the glucose biosensor [35]. XPS analysis was conducted on a PHI 5600 spectrometer with a hemispherical energy analyzer using an aluminum (Kα) source of 1487 eV at 100 Watts. The pressure in the analysis chamber during XPS analysis was maintained in the low range of 10^−9^ Torr. The analyzed area was around 1 mm^2^. All the spectra were recorded at a 54° take-off angle, with a 1.0 eV step. The spectra were further corrected using a carbon signal (C1s) at 284.5 eV and analyzed using Casa-XPS software version 2.3.18. The Shirley method was used to extract the background necessary for the curve fitting.

## 3. Results and Discussion

### 3.1. Surface Characterization

#### 3.1.1. Analysis of GOX Immobilization via FT-IR Spectroscopy

**Figure 2:**
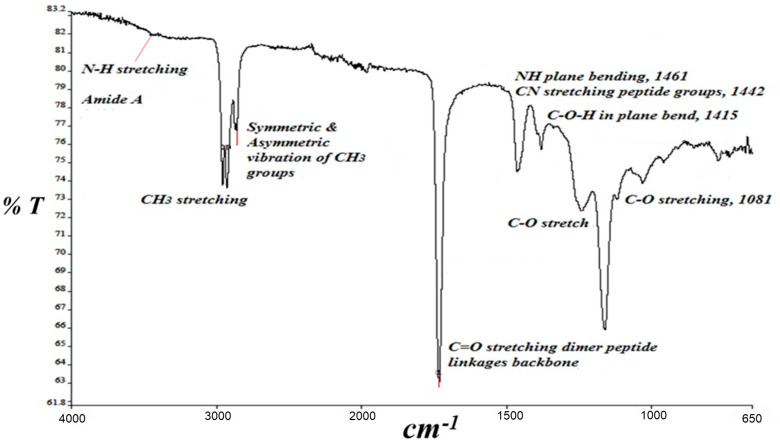
FT-IR spectra for PA-PPE-GOX.

Using FT-IR spectroscopy, we observed the presence of the amide I band (an overlapping spectrum of α-helices, β-sheets, turns, and random coils, which form the basic structure of the protein) [36] (Figure 2) at 1650 cm^−1^ and 1715 cm^−1^ caused by C=O stretching vibrations, which identified the C=O stretching vibrations of the peptide linkages in the GOX backbone at 1600 cm^−1^ to 1700 cm^−1^ and 1650 cm^−1^ to 1750 cm^−1^ [29,37]. Furthermore, the amide band II peak at 1462 cm^−1^ was observed due to the combination of N-H in-plane bending and C-N stretching linkages for the peptide groups [37,38,39]. There was another small band, amide band III, at 1239 cm^−1^, which correlates well with the Delfino data, 2013 [40]. In addition, we found two wide bands in the region 3400 to 2900 cm^−1^, representing the amide A and bond linkages to -CH_3_ stretching at 3400 cm^−1^ and 2958 cm^−1^, respectively [25]. Therefore, the FT-IR spectra indicate the successful immobilization of GOX on the PA-PPE surface.

#### 3.1.2. Specific Surface Area

The specific surface area of the sample was calculated by the Brunauer–Emmett–Teller (BET) [41] surface adsorption method through the accelerated surface area and porosimetry system (ASAP) 2020 Sorptometer from Micromeritics by measuring the N_2_ adsorption on the surface of the sample. The sample was degassed at 150 °C for 70 min at 10 mm Hg to remove any remaining solvent and ensure complete dryness and emptiness of the pores. Hence, the amount of adsorbed N_2_ at a bath temperature of 76 K was obtained. The BET surface area report showed 5.07 m^2^ per gram of material. The geometric surface area for WE is 0.03141 cm^2^. The Brunauer–Emmett–Teller (BET) surface area is 12.675 cm^2^/g, with a pore size of 92.607 Å and pore volume of 0.016736 cm^3^/g. 

#### 3.1.3. Analysis of GOX Immobilization via XPS

**Figure 3:**
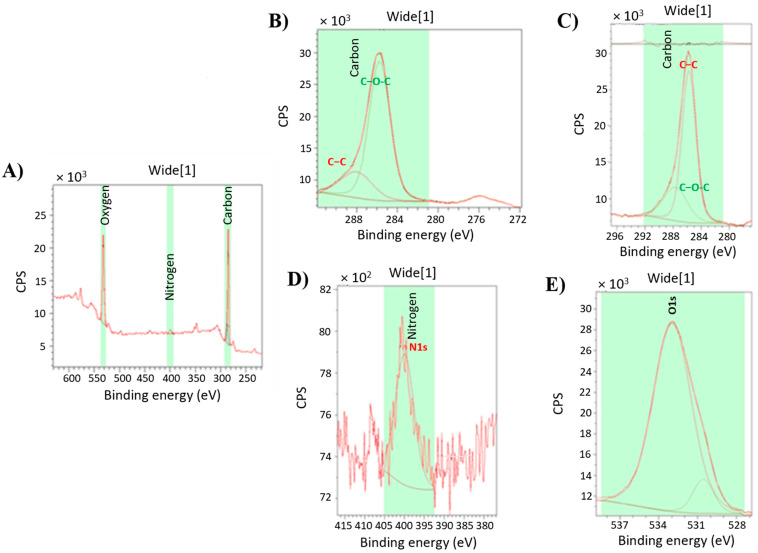
XPS for GOX (**A**) found C(1s) (**B**,**C**), N(1s) (**D**), and O(1s) (**E**) spectra.

XPS studies were conducted on the PA-PPE-GOX sensor to understand GOX immobilization [36]. The peaks of N and O from the XPS survey scan in Figure 3A indicate the immobilization of GOX on the carbon electrode. The XPS high-resolution scan data in Figure 3 illustrate that the data for C was fitted by a mix of pure C(1s), C-C, and C-O-C bonds in the following proportions. The peak of C(1s) shifted from 284 eV and was focused around 285.8 eV, corresponding to the overlap of the C-C, C-H, and C=C bonds [42,43]. A C(1s) centered peak was observed around 288 eV with two binding energies, 288.3 (N-C-O bond) and 286.3 (C-O bond), which can be assigned to overlap groups such as [R-CH2-NH-(C*O)-] and [(R-CH2*-NH-(CO)-], respectively [42], and presents the overlap of C=N, C≡N and C-O bonds [43]. These findings are consistent with previous XPS characterizations [44,45,46]. For instance, Li et al. reported two significant changes in the peaks obtained by the binding energy for immobilized GOX on the film surface. The first change corresponded to the reduction in the intensity of the carboxyl group near 288.7 eV, and the second change corresponded to an increase in intensity at the peak of the peptide union at 287.8 eV [44]. Later, Dementjev et al. found a peak with a union energy at 287.9 eV [46] that was identified as C(1s), indicating the interaction between C and N, specifically for carbon atoms (four bonds) that have a single or double bond with nitrogen atoms (C-4N). 

In the binding energy of the N(1s) spectrum (Figure 3D), we found a centered peak at 400 eV, which corresponded to the pure N(1s) peak at 399 eV [42]. The N(1s) spectra core-level found the following binding energies at different peaks and was attributed to their respective unions, such as 398.3 eV (overlap N^1^ sp^3^ as N-C and C-NH_2_ bonds) [43,46,47], 399.1 eV (overlap N≡C and C=N-C bonds) [43], 399.9 eV (N=C bond) [43], 400.6 eV (N-C sp^2^ bonding) [47], and 400.8 eV [NO (N4)] [45]. These data confirm the presence of organic molecules, due to the configurations of the N(1s) and C(1s) spectra, in the surface of the biosensor and support the immobilization of GOX. The centered peaks overlapping binding energy for 398.5 eV, 398.8 eV, 399.1 eV, and 400.7 eV were attributed to the (C-NH_2_ bond) [46], (N-C sp3 bonding) [47], (C=N-C bond) [46], and overlapping N-C sp2 bonding and NH_2_ groups [43], respectively. All of them contributed to identifying the different bonds between the N(1s) and C(1s) as a part of the configurations of the carbon nitride compounds. The XPS data we obtained, especially regarding the changes in the spectra of C(1s) and N(1s), related to the chemical group characteristics of the proteins, indicated that GOX was successfully immobilized in our PA-PPE-GOX biosensor. 

### 3.2. Electrochemical Characterization 

**Figure 4:**
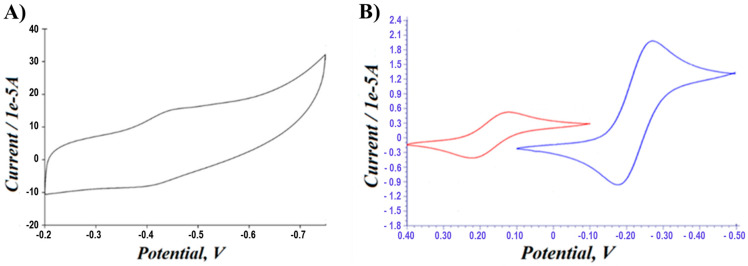
(**A**) CV response of PPE-PA-GOX in 0.01 M PBS at a scan rate of 50 mV/s. (**B**) CV response comparison for PPE-PA-GOX between 5 mM potassium ferricyanide (anionic probe-left side) and 5 mM ruthenium-hexamine chloride (cationic probe-right side).

After immobilizing GOX onto the PA-PPE, the electrochemical characterization was performed in 0.01 M PBS under N_2_. The CV profile for PA-PPE-GOX exhibited a pair of well-defined and nearly symmetric redox peaks with a formal potential of −0.44 V (see Figure 4A) close to the standard electrode potential of GOX [48], which was similar to previous reports [23]. In addition, we compared the CV responses for PPE-PA-GOX at the scan rate of 0.050 V/s between 5 mM potassium ferricyanide, an anionic probe (left), and 5 mM ruthenium-hexamine chloride 5 mM, which exhibited a cationic probe graphics (Figure 4B, right). Two different probes toward the same electrode explained the electron transfer involved and the interaction between the electrodes (negative charge) and the electrostatic interaction between the electrode and probe molecules (specific potential for oxidation and reduction).

The difference in the potential between the peaks of the reduction (*E_pc_*) and oxidation (*E_pa_*) curves was Δ*E_p_* = (*E_pa_* − *E_pc_*) = 0.0599 V (a value that is close to the theoretical value of 0.059 V for the ferrocene redox pair) for all the scan rates in our experiments. The peak current ratio (*i_pa_/i_pc_*) was equal to 1.01, and the formal electrode potential for a redox process was Eo’ = 0.43145. The electrochemical response of GOX immobilized onto the PA-PPE is attributed to the direct electron transfer of GOX for the conversion of FAD/FADH_2_ [23,49,50]. The direct electron transfer reaction of the GOX/FAD redox reaction involves two electrons coupled with two protons, as shown by the following chemical reaction:

*GOX*/*FAD* + 2*e*^−^ + 2*H*^+^ = *GOX*/*FADH*_2_

**Figure 5:**
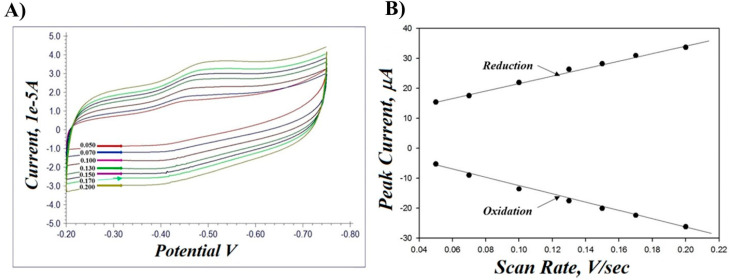
(**A**) CV response of PA-PPE immobilized with GOX in 0.01 M PBS at a scan rate of 50–200 mV/s. (**B**) Plots of anodic (*i*_pa_) and cathodic (*i*_pc_) peak current versus scan rates.

We also investigated the electrochemical responses at different scan rates. The scan rate effect on the PA-PPE-GOX electrodes is shown in Figure 5. When the scan rates (v) increased from 50 to 200 mV/s (Figure 5A), the anodic and cathodic peak currents changed appreciably and increased with respect to the change in the scan rates and exhibited a good linear relationship between both anodic and cathodic current and scan rates (Figure 5B), indicating a surface-diffusion controlled redox electrode process. The relationship between the log peak current versus the log scan rate was linear with a slope of 0.561714 (a = transfer coefficient).

The electron transfer rate constant, k_s_, on the PA-PPE-GOX electrode, was calculated with the Laviron equation [51]:

*Log k_s_* = *α log* (1 − *α*) + (1 − *α*) *logα* − *log* (*RT*/*nF ν*) − *α* (1 − *α*) *nF*Δ*Ep*/2.3*RT*

where the calculated charge transfer coefficient (*α*) is 0.5617 with two electrons transfer (*n* = 2), *R* is the universal gas constant (8.314 J mol^−1^K^−1^), *T* is the room temperature in Kelvin degrees (298 °K), *F* is the Faraday constant (9.64853 × 10^4^ °C), and *v* is the scan rate.

The electron transfer rate constant, *k_s_*, was calculated to be 3.363 s^−1^, which was higher than the *k_s_* reported for carbon nanostructured materials, such as a K_s_ of 1.69 s^−1^ from Janegitz et al. [24], a K_s_ of 2.83 s^−1^ from Kang et al. [48], a K_s_ of 3.273 s^−1^ from Hua et al. [52], and a K_s_ of 1.713 s^−1^ and 1.12 s^−1^ from Razmi et al. [53], and gold nanoparticle incorporated matrices (a Ks of 1.713 s^−1^ from Wu et al. [54] and a Ks of 2.2 s^−1^ from Zhang et al. [55]). From this, it is evident that the carbonyl functionalities and edge plane-like sites formed during the pre-anodization process at PPE play an important role in improving the electron transfer communication between the redox centers of GOX and the electrode. 

The surface average concentration of the electroactive GOX (*Γ*) value was calculated from the equation *Γ* = *Q/nFA,* where (*Q*) is the charge involved in the reaction, (*n*) is the number of electrons transferred, (*F*) is the Faraday constant, and (*A*) is the area of the WE. According to the equation, *Γ* of our system was calculated to be Γ = 8.30013 × 10^−9^ mol/cm^2^, which was higher than other published values, such as *Γ* = 2.56 × 10^−10^ mol/cm^2^ [55], *Γ* = 5.1 × 10^−11^ mol/cm^2^ [37], *Γ* = 1.12 × 10^−9^ mol/cm^2^ [49], *Γ* = 1.8 × 10^−9^ mol/cm^2^ [53], and *Γ* = 4.65 ± 0.76 × 10^−10^ mol/cm^2^ [56] implying that the higher heterogeneous direct electron transfer rate constant is directly influenced by the multilayer coverage of GOX.

### 3.3. Square Wave Voltammetry (SWV) Analysis

**Figure 6:**
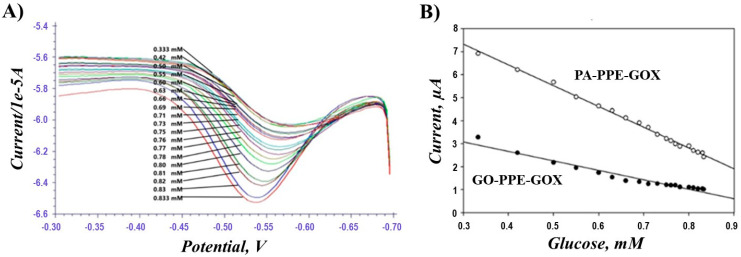
Square wave voltammogram (SWV) for different concentrations of glucose at PA-PPE-GOX (**A**) and the corresponding calibration curve for PA-PPE-GOX and GO-PPE-GOX (**B**).

Additionally, the electrocatalytic activity of the PA-PPE-GOX toward glucose was studied by conducting SWV experiments with different concentrations of glucose ranging from 0.2 mM to 0.84 mM in 0.1 M PBS solution under N_2_. The successive addition of glucose resulted in a gradual decrease in the reduction current (Figure 6A), which was linearly proportional to the increased concentration of glucose. This trend can be explained by the fact that the addition of glucose triggers the enzyme-catalyzed reaction between GOX and glucose by the formation of FADH_2_ from FAD, resulting in a subsequent decrease in the cathodic peak current [24,57]. This reaction causes a decrease in the amount of oxidized GOX on the PA-PPE electrode and reduces the electrode reduction current. To demonstrate the increased sensitivity of the newly developed electrode, PA-PPE-GOX, toward glucose detection, we compared its response with the current response developed for GOX immobilized on graphene oxide through the EDC/NHS cross-coupling method, GO-PPE-GOX. As can be seen from the calibration plots of PA-PPE-GOX and GO-PPE-GOX (Figure 6B), the pre-anodized electrode exhibited a 2.612 times higher current response than the GO-modified electrodes. The improved electrocatalytic activity at PA-PPE-GOX again points toward better electron transfer communication between GOX and the electrodes at the electrochemically activated or pre-anodized electrodes than the GO-modified electrodes. 

### 3.4. Chronoamperometry Analysis (CA)

**Figure 7:**
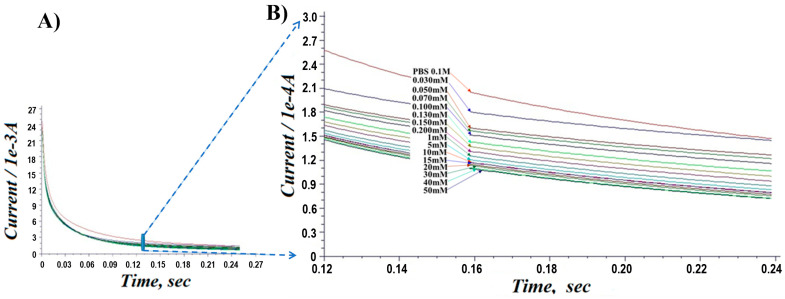
Chronoamperometric responses for PA-PPE-GOX toward different concentrations of glucose (30 µM to 50 mM). Section of (**A**) is zoomed in as (**B**).

**Figure 8:**
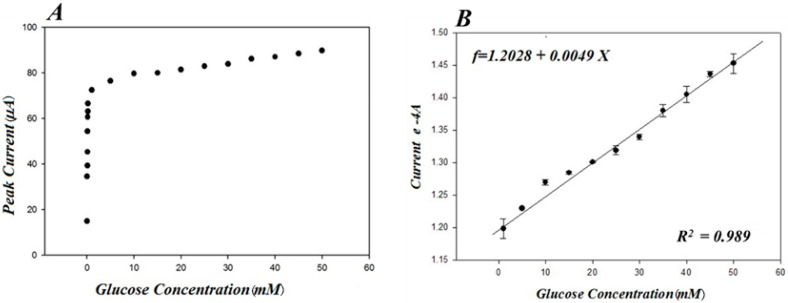
Peak current responses versus different glucose concentrations by the chronoamperometry method (**A**) and their corresponding linearity range from 1 mM through 50 mM (**B**).

The electrocatalytic performance of PA-PPE-GOX was further characterized by conducting chronoamperometry experiments for the reduction of different concentrations of glucose in 0.01 M PBS (pH 7.4), ranging from 30 μM to 50 mM. Figure 7 depicts the CA responses of glucose reduction at different concentrations of glucose at −0.55 V. The negative operating potential for the detection of glucose eliminates the interference from other common electroactive species, such as ascorbic acid, uric acid, and dopamine, that can be present in real blood samples, which is another advantage of our direct electron transfer glucose biosensor. As the concentration of glucose increased, there was a shift from the linearity between the concentration and current (Figure 8A), exhibiting typical Michaelis-Menten kinetics [58,59]. The Lineweaver–Burk plot [57], a double reciprocal graphical representation of the enzyme kinetics, shows a linear curve graphic based on the following equation:

1/*v* = (1/*v_max_*) + {(*K_m_*/*v_max_*) (1/[*S*]),

where *v* is the initial reaction rate at a given substrate concentration, *v_max_* is a constant reaction rate, *K_m_* is the Michaelis–Menten constant, and [*S*] is the substrate concentration. Vmax = 0.021714, m = 1.38343, and b = −46.05323, which corresponds to the linear equation *y* = *b + mx*, with a direct correlation between the current *1/v* (*1/mA*) and the glucose concentration *1*/[*S*] (*1/mM*) [59].

The calculated *K_m_* was found to be 0.03 mM, which is lower than that of the GOX immobilized on graphene quantum dots (*K_m_* of 0.76 mM) [53], the reduced graphene oxide (*K_m_* of 0.215 mM) [60], the poly (p-phenylenediamine)-based nanocomposite (*K_m_* of 0.42 mM) [56], and the pre-anodized screen-printed carbon electrodes (*K_m_* of 1.07 mM) [23]. A lower *K_m_* means a higher enzymatic activity of the immobilized GOX at PA-PPE. Thus, the results suggest that the presented PA-PPE-GOX had a high affinity toward glucose, with a limit of detection as low as 5.4 mg/dL (0.3 mM). Figure 8B shows a linearity range for glucose of 0.3 mM through 50 mM (i.e., 5.4–900 mg/dL) with an R^2^ value of 0.989, which includes the normal range of blood glucose in humans as well the abnormal range values in hypoglycemia or hyperglycemia. Most commercial glucometers can only achieve quantitative measurement of glucose in a limited range of operation.

### 3.5. Analytical Validation

**Figure 9:**
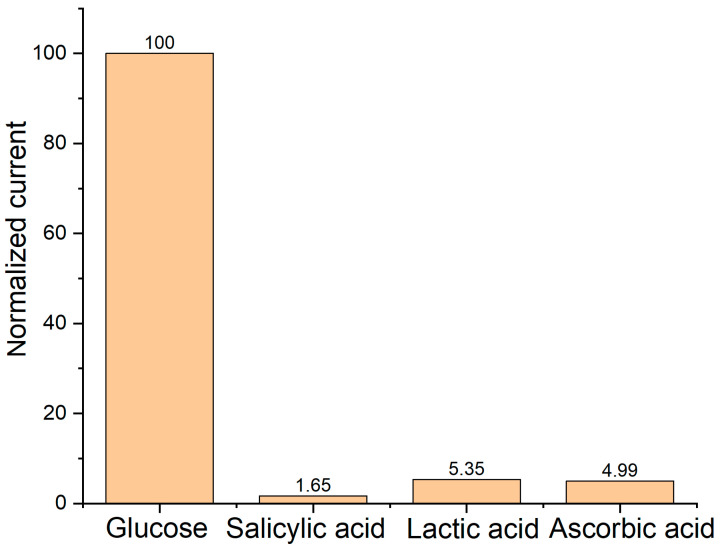
Specificity investigation by testing different interfering species. *E*, 0.5 V.

There are substances in the blood that are considered to be interfering species and can interfere with glucose readings in biosensors. We conducted specificity tests using the amperometric *i-t* technique to validate the specificity of the biosensor PA-PPE-GOX on glucose, as well as ascorbic acid (AA), salicylic acid (SA), and lactic acid (LA) as interfering species. The concentrations of the interfering species were adjusted to a similar concentration as glucose (0.1 M). The amperometric readings are shown in Figure 9, and the currents of the interfering species were very low. We calculated the current ratios between glucose and the interfering species, which were 1.65%, 5.35%, and 4.99% for SA, LA, and AA, respectively. The current ratios between glucose and the interfering species allowed us to infer that our sensor shows great specificity for glucose. The current values of the interfering species represent a higher concentration (0.1 M) than the concentrations that these species can obtain in blood. Therefore, we observed a minimum current response of the interfering species with respect to glucose, indicating high selectivity of the biosensor toward glucose.

The validation for the electrode PA-PPE-GOX towards glucose was carried out through analytical recovery experiments, which were performed with different samples of whole blood, and the percentage of recovery was calculated from the data. The recovery values ranged from 90% to 101%, with an average of 95%, which are in the acceptable range in bioanalytical chemistry. In addition, three human whole blood samples were measured using our method, and the results are shown in Figure S1 and Table S1 (in the Supplementary Materials) which were consistent with the measured values from a commercial glucometer. To review the stability, different electrodes were tested for 3 months. The PA-PPE-GOX-glucose showed an error percentage of 3.76%, showing that the electrodes have good shelf life and stability. 

## 4. Conclusions

Herein, we developed a simple method for direct electron transfer of glucose oxidase on a disposable pre-anodized paper-carbon electrode modified through zero-length cross-linkers and demonstrated its application for glucose biosensors. FT-IR, XPS, and electrochemical methods were used to characterize the immobilization of GOX. The DET-based glucose biosensor has the following significant features:

(1) The direct electron transfer constant, *K_s_* 3.363 s^−1^, is much higher than that demonstrated in previous studies of carbon nanostructured materials [24,48,52,53] and gold nanoparticle [54,55] incorporated matrices and comparable with SPCE-Nafion^®^ film, with a rapid detection time of less than 10 s.
(2) The surface average concentration of electroactive GOX (*Γ*) value was calculated to be 8.30013 × 10^−9^ mol/cm^2^, which was higher than other published values [48,53,55,56]. This indicates that the higher heterogeneous direct electron transfer rate constant is directly influenced by the multilayer coverage of GOX.
(3) The DET-based glucose detection at PA-PPE-GOX showed higher electrocatalytic activity (2.61-fold) than a graphene oxide composite modified electrode.
(4) PA-PPE-GOX has a higher affinity for glucose, *K_m_* 0.03 mM, with a wide range to detect glucose from 5.4 mg/dL to 900 mg/dL, which involves the glucose human range to measure diabetes variability in hypoglycemic and hyperglycemic states. In contrast, most commercially available glucometers can only achieve quantitative measurement of glucose in a limited range of operation. Our PA-PPE-GOX-glucose biosensor meets both recommendations addressed by the FDA and SMBG to minimize the hypoglycemia state and maximize euglycemia [61].
(5) Furthermore, our electrode (PPE-PA-GOX-glucose) showed remarkable stability and selectivity. The use of the negative operating potential eliminates the interference from ascorbic acid, uric acid, and dopamine.

This DET method can be used to immobilize other proteins and enzymes to achieve a higher electrocatalytic activity in various bioassays [62,63,64,65,66]. Our next step in our future work is to conduct clinical validation, especially for self-monitoring of blood glucose (SMBG).

## Data Availability

The data are available on request.

## Supplementary Materials

The following supporting information can be downloaded at: https://www.mdpi.com/article/10.3390/bios13050566/s1, Figure S1: Measurement of human whole blood samples using the paper-based glucose biosensor via direct electron transfer; Table S1: Glucose measurement results of human whole blood samples (mM).
