# Supplementary material for: Direct Electron Transfer of Glucose Oxidase on Pre-Anodized Paper/Carbon Electrodes Modified through Zero-Length Cross-Linkers for Glucose Biosensors
Source: Biosensors (Basel). 2023 May 22;13(5):566. doi: 10.3390/bios13050566 (PMC10216203; doi:10.3390/bios13050566)
Supplement: Supplementary file 1 [file biosensors-13-00566-s001.zip › biosensors-2289993-supplementary.pdf]

Supplementary Information

Direct Electron Transfer of Glucose Oxidase on Pre-Anodized Paper/Carbon Electrodes Modified through Zero-Length Cross-Linkers for Glucose Biosensors

Gilberto Henao-Pabon [1,†], Ning Gao [2,†], K. Sudhakara Prasad [3,4,†] and XiuJun Li [1,3,5,*]

[1] Biomedical Engineering, University of Texas at El Paso, 500 W University Ave, El Paso, TX 79968, USA
[2] Independent Researcher, 206 via Morella, Encinitas, CA 92024, USA
[3] Department of Chemistry & Biochemistry, University of Texas at El Paso, 500 W University Ave, El Paso, TX 79968, USA
[4] Yenepoya Research Centre, Yenepoya University, Mangalore 575018, Karnataka, India
[5] Forensic Science & Environmental Science and Engineering, 500 W University Ave, El Paso, TX 79968, USA
* Correspondence: xli4@utep.edu
† These authors contributed equally to this work.

Measurement of spiked human whole blood samples

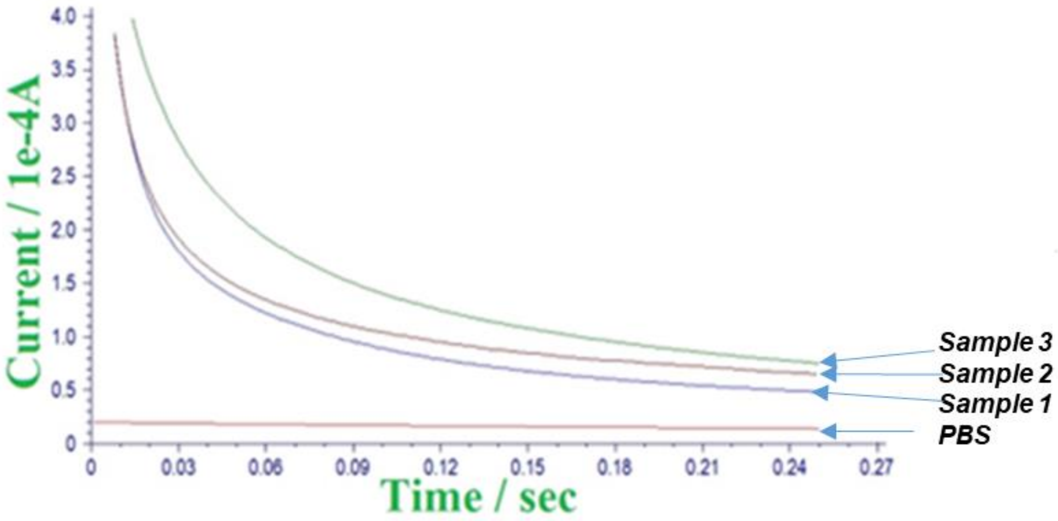

Figure S1. Measurement of human whole blood samples using the paper-based glucose biosensor via direct electron transfer.

Table S1. Glucose measurement results of human whole blood samples (mM).

Glucose	Mean	Mean± Std. Dev.	Std. Error
Sample No. 1	5.9533	5.9533± 0.1721	0.0994
Sample No. 2	7.8353	7.8353± 0.1780	0.1028
Sample No. 3	9.05	9.050± 0.1651	0.0953
